# A rare constellation of bilateral progressive visual and auditory loss in neurofibromatosis type 2: a multimodal diagnostic approach

**DOI:** 10.1097/MS9.0000000000004545

**Published:** 2025-12-18

**Authors:** Shreya Khandelwal, Aman Kumar, Shruti Sharma, Amneet Kaur, Arun Kumar Bandyopadhyay

**Affiliations:** a Department of Ophthalmology, B.P. Koirala Institute of Health Sciences (BPKIHS), Dharan, Sunsari, Nepal; b Department of Radiology, MGM Medical College, India; c Department of Ophthalmology, MGM Medical College, India

**Keywords:** bilateral vestibular schwannoma, case report, neurofibromatosis type 2, OCT, optic atrophy, sensorineural hearing loss, spinal ependymoma

## Abstract

**Background:**

Neurofibromatosis Type 2 (NF2) is a rare autosomal dominant disorder characterized by bilateral vestibular schwannomas and a spectrum of central and peripheral nervous system tumors. Early diagnosis can be challenging, particularly in the absence of classic skin findings or in resource-constrained settings.

**Case presentation:**

We present the case of an 18-year-old female who developed progressive bilateral vision loss, hearing impairment, and a longstanding posterior cervical mass. Ophthalmic evaluation revealed bilateral optic atrophy, with profound visual impairment. Audiological testing confirmed bilateral sensorineural hearing loss. MRI of the brain and spine demonstrated bilateral cerebellopontine angle tumors consistent with vestibular schwannomas, along with extensive craniospinal tumors including spinal ependymoma and multiple schwannomas. Optical coherence tomography (OCT) revealed significant thinning of the retinal nerve fiber layer, consistent with optic neuropathy. The constellation of findings confirmed a diagnosis of Neurofibromatosis Type 2.

**Clinical discussion:**

The simultaneous occurrence of optic atrophy, bilateral vestibular schwannomas, and multiple spinal tumors illustrates the classic but complex presentation of NF2. This case highlights how multimodal diagnostic tools – including neuroimaging, audiological assessment, and OCT – play a vital role in confirming the diagnosis in patients with progressive multisystem neurological deficits.

**Conclusion:**

This case underscores the importance of considering NF2 in young patients with simultaneous multisystem neurologic deficits, even in the absence of cutaneous stigmata. A high index of clinical suspicion, supported by multimodal imaging, is essential for early diagnosis and appropriate multidisciplinary management in such complex presentations.

## Introduction

Neurofibromatosis type 2 (NF2) is an uncommon autosomal dominant tumor-predisposition syndrome caused by pathogenic variants in the *NF2* gene, which encodes merlin, a cytoskeletal tumor suppressor protein^[1]^. The disorder is classically defined by the presence of bilateral vestibular schwannomas and is frequently associated with additional intracranial and intraspinal tumors such as meningiomas, ependymomas, and peripheral nerve schwannomas^[2]^. While NF1 and NF2 are sometimes conflated, they are genetically and clinically distinct: NF1 results from *NF1* gene mutations and is typified by café-au-lait macules, cutaneous neurofibromas, and optic pathway gliomas, whereas NF2 primarily manifests with vestibular schwannomas, meningiomas, and ocular abnormalities including early cataract, epiretinal membranes, and, less commonly, optic nerve involvement^[3]^.

The estimated prevalence of NF2 ranges from 1 in 25 000 to 1 in 60 000, with a birth incidence of approximately 1 in 33 000–40 000, though figures vary across populations^[4,5]^. Despite its rarity, NF2 exhibits striking phenotypic heterogeneity. Progressive sensorineural hearing loss remains the most characteristic early feature, yet initial manifestations may also include visual dysfunction from optic nerve compression or atrophy, cranial neuropathies, or spinal cord compromise^[3]^. These atypical presentations may delay diagnosis, particularly in resource-limited settings where timely access to neuroimaging and genetic testing is constrained.

Here, we report the case of an 18-year-old female who presented with simultaneous progressive bilateral visual loss, profound sensorineural hearing impairment, and a chronic posterior cervical mass, ultimately diagnosed as NF2 following detailed neuro-ophthalmic evaluation and comprehensive craniospinal imaging. This case is notable for its synchronous optic and auditory dysfunction at presentation – an uncommon phenotype – and highlights the importance of maintaining a high index of suspicion for NF2 in young patients with multisystem neurological deficits. It further illustrates the pivotal role of multimodal imaging, particularly MRI, in delineating tumor burden, informing prognosis, and guiding multidisciplinary management. This article has been prepared in accordance with the TITAN checklist and the CARE guidelines to ensure completeness, transparency, and quality in reporting^[6,7]^.

## Presenting complaints

**Figure 1.**
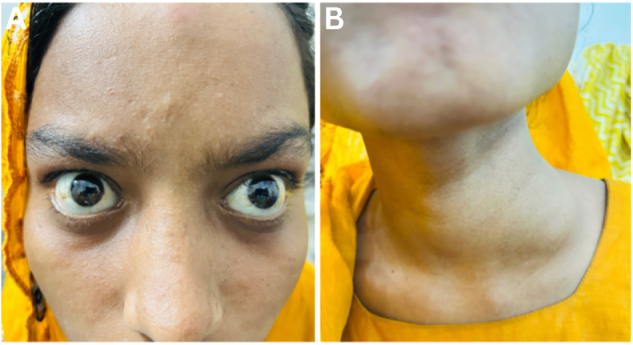
(A) Clinical photograph showing the patient in primary gaze. (B) Close-up view of the left lateral neck demonstrating a large, non-tender, lobulated swelling occupying the posterior triangle. The overlying skin appears normal, with no visible pulsation or discoloration.

An 18-year-old female presented with progressive, painless loss of vision in both eyes for 1 month, accompanied by hearing impairment for 3 months and a painless swelling on the left side of her neck persisting for 3 years as shown in Figure 1.

## History of present illness

The patient first noticed gradual blurring of vision in both eyes 1 month prior, which progressed to only perception of light bilaterally. There was no pain, redness, photophobia, transient obscurations, flashes, floaters, or diplopia.

Approximately 3 months earlier, she developed bilateral, progressive hearing loss with tinnitus. She reported difficulty following conversations in noisy environments and required increased device volume, with occasional imbalance but no vertigo, otalgia, or discharge.

The neck swelling was first noticed 3 years ago. It was located in the left posterior triangle of the neck, was painless, and had gradually increased in size over time. There was no associated fever, weight loss, night sweats, or overlying skin changes. She did not report dysphagia, hoarseness, or respiratory symptoms.

She denied headaches, nausea, vomiting, or other symptoms suggestive of raised intracranial pressure. There was no history of limb weakness, sensory disturbances, or bladder/bowel dysfunction.

### Past medical and surgical history

She had no prior history of ocular disease, head trauma, chronic illnesses such as diabetes or hypertension, or previous surgeries. She was not on any regular medications and had no known drug allergies.

**HIGHLIGHTS**

- This case report documents a rare and atypical presentation of Neurofibromatosis Type 2 in a young female, marked by simultaneous progressive bilateral visual and auditory loss as the initial clinical manifestation.
- The absence of cutaneous stigmata and delayed presentation posed a significant diagnostic challenge, emphasizing the need for heightened clinical suspicion in multisystem neurologic presentations.
- Multimodal imaging – including MRI of the brain and spine and optical coherence tomography – was pivotal in confirming the diagnosis and delineating the full extent of craniospinal tumor involvement.
- The case underscores the importance of a multidisciplinary diagnostic approach in resource-limited settings where early genetic testing and neuroimaging may not be readily accessible.
- This report contributes to the growing body of literature on phenotypic variability in NF2 and reinforces the value of early neuro-ophthalmic assessment in guiding diagnosis and management in syndromic neurocutaneous disorders.

### Family history

There was no family history of similar neurological or sensory complaints, hearing loss, vision problems, neurocutaneous disorders, or malignancies.

### Social history

She was a student, with no history of smoking, alcohol, or illicit drug use. There was no significant occupational or environmental exposure to noise or toxins. She had not traveled recently and reported no exposure to tuberculosis or other infectious diseases.

She denied constitutional symptoms such as fever, malaise, or weight loss. There were no respiratory, gastrointestinal, genitourinary, or musculoskeletal complaints.

## Examination

On general assessment, the patient was alert, oriented, and hemodynamically stable. No skin abnormalities were identified; specifically, there were no café-au-lait macules, cutaneous neurofibromas, or other neurocutaneous stigmata.

Ophthalmic examination revealed no obvious proptosis on inspection. Hertel exophthalmometry measured 18 mm in the right eye and 19 mm in the left eye. There was no periorbital edema, chemosis, or globe displacement in other planes. Extraocular movements were full in all directions, without restriction or diplopia. Visual acuity was reduced to perception of light in both eyes. The right pupil reacted sluggishly, while the left demonstrated a relative afferent pupillary defect. The anterior segment was unremarkable, with normal intraocular pressures (16 mm Hg right, 18 mm Hg left). Fundus evaluation showed bilateral optic disc pallor, more marked temporally, with mild cupping but no papilledema, hemorrhages, or exudates. Optical coherence tomography confirmed marked thinning of the peripapillary retinal nerve fiber layer. Macular function (Amsler grid), color vision, and perimetry could not be reliably assessed, consistent with severe bilateral optic neuropathy.

ENT examination was notable for a very large, firm, non-tender, mobile mass measuring approximately 12 × 7 cm, occupying the entire left posterior triangle of the neck. The overlying skin was normal, and there was no transillumination or bruit. The mass extended anteriorly beyond the sternocleidomastoid. Otoscopic examination revealed intact, healthy tympanic membranes bilaterally. Pure tone audiometry demonstrated symmetric, moderate-to-severe sensorineural hearing loss across all frequencies, with significantly reduced speech discrimination scores. Vestibular function was assessed with bedside maneuvers, including the Dix-Hallpike and head impulse tests, both of which were negative for positional vertigo or nystagmus. The patient reported mild imbalance, and Romberg’s test was mildly positive, suggesting vestibulocochlear nerve involvement. There was no facial asymmetry or weakness, and corneal reflexes were preserved.

Neurological examination showed no additional cranial nerve deficits; gag reflex and palate elevation were normal, tongue movements intact. Motor and sensory examinations were normal, with preserved reflexes and flexor plantar responses. Coordination was intact, though tandem gait was mildly unsteady. Systemic examination was unremarkable.

Systemic examination, including cardiovascular, respiratory, and abdominal systems, was unremarkable. There were no constitutional symptoms or evidence of systemic disease.

The combination of bilateral optic neuropathy, profound sensorineural hearing loss, vestibular dysfunction, and a cervical mass mandated a broad differential diagnosis.

For the visual loss, demyelinating optic neuritis was considered but excluded due to absence of acute episodes, poor recovery, and established optic atrophy. Compressive optic neuropathy from meningioma was a possibility, yet imaging demonstrated multiple tumors rather than a solitary lesion. Mitochondrial optic neuropathy (e.g., Leber hereditary optic neuropathy) was unlikely in the absence of maternal inheritance and systemic features. Glaucoma was excluded by normal intraocular pressures and optic disc morphology, while optic pathway glioma, more typical of NF1, was not supported by the imaging pattern.

For the auditory findings, autoimmune inner ear disease was considered but the symmetry, chronicity, and lack of inflammatory signs argued against it. Congenital/hereditary SNHL was possible but atypical given the late presentation with concurrent optic atrophy and mass lesion. Ototoxicity was excluded by history, and demyelinating disease (such as MS) could not account for the multiplicity of cranial nerve tumors seen on MRI.

Ultimately, the presence of bilateral vestibular schwannomas on MRI, together with bilateral SNHL, optic atrophy, and an additional cervical schwannoma, established the diagnosis of Neurofibromatosis type 2. This clinico–radiologic correlation was decisive and rendered alternative diagnoses unlikely.

## Management

**Figure 2.**
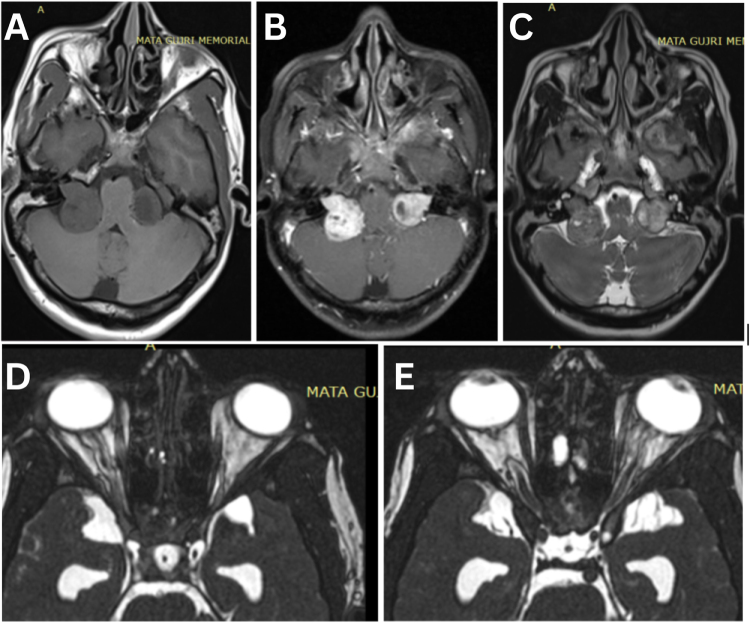
(A) Axial T1-weighted MRI of the brain demonstrating lobulated iso- to hypointense soft tissue masses in the bilateral cerebellopontine angle regions extending into the internal auditory canals. (B) Axial T1-weighted post-contrast MRI showing vivid enhancement of the solid components of the bilateral CP angle tumors, with non-enhancing cystic areas within the lesions. (C) Axial T2-weighted MRI illustrating heterogeneously hyperintense signal of the CP angle masses with cystic components and associated compression of adjacent structures. (D) Axial CISS MRI sequence showing thinning and increased signal intensity of the right intraconal optic nerve, with prominence of the perineural cerebrospinal fluid space, consistent with optic nerve atrophy. (E) Axial CISS MRI sequence demonstrating similar atrophic changes of the left intraconal optic nerve, with marked perineural CSF prominence and loss of normal nerve caliber.

**Figure 3.**
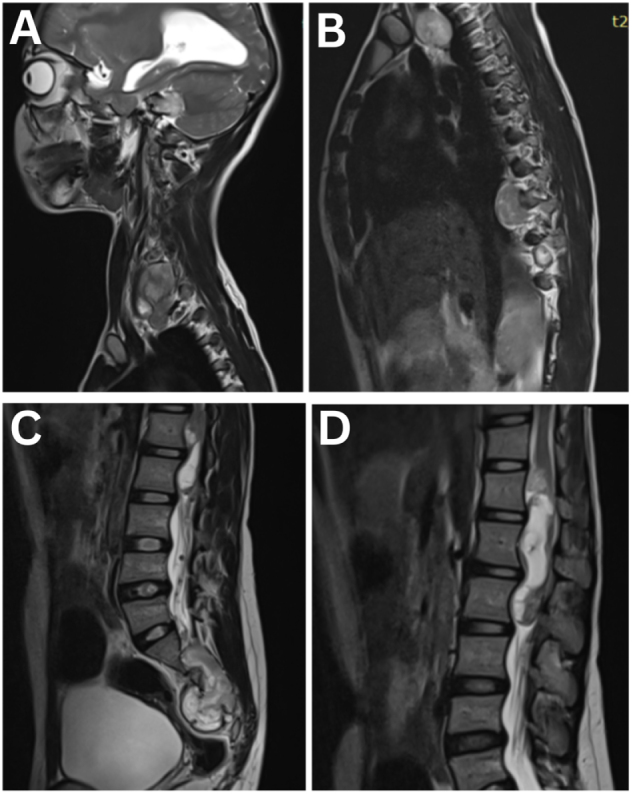
(A) Sagittal T2-weighted MRI of the cervical spine demonstrating a lobulated, heterogeneous lesion extending into the neural foramina along the exiting nerve roots, with features suggestive of a nerve sheath tumor and extramedullary intradural extension at the C6–D1 level. (B) Sagittal T2-weighted MRI of the dorsal spine showing similar lobulated lesions with intradural extramedullary extension at the D9–D10 level. (C) Sagittal T2-weighted MRI of the sacrococcygeal spine demonstrating extension of the lesion into the neural foramina at the sacrococcygeal level. (D) Sagittal T2-weighted MRI of the lumbosacral spine showing an intramedullary lesion involving the terminal spinal cord and filum terminale, extending from D12 to L2, resulting in fusiform dilatation of the cord and scalloping of the posterior vertebral margins.

**Figure 4.**
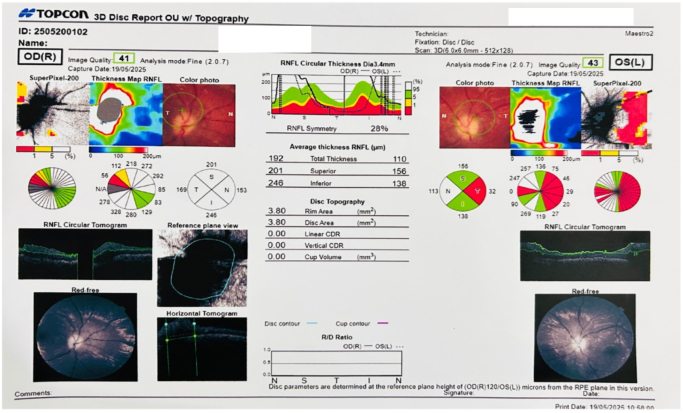
Optical coherence tomography (OCT) image demonstrating marked thinning of the peripapillary retinal nerve fiber layer (RNFL) in both eyes. Overall retinal thickness is reduced, consistent with advanced optic nerve atrophy.

The patient underwent a comprehensive panel of laboratory and imaging investigations to establish the extent and etiology of her multisystem involvement. Baseline hematological and biochemical tests, including complete blood count, renal and liver function, electrolytes, thyroid profile, and glycemic parameters, were within normal limits. MRI of the brain (Fig. 2A–C) demonstrated bilateral cerebellopontine angle lobulated lesions extending into the internal auditory canals, with cystic components, heterogeneous contrast enhancement, and foci of calcification, consistent with vestibular schwannomas. The optic nerves showed increased signal intensity, thinning, and prominent perineural cerebrospinal fluid spaces, indicating advanced optic atrophy consistent with optic neuropathy (Fig. 2D and E). Notably, there was associated hydrocephalus with ventricular dilatation. MRI spine screening (Fig. 3A–C) revealed multiple lobulated lesions along the exiting nerve roots with intradural extramedullary extension at C6–D1, D9–D10, and the sacrococcygeal levels, as well as an intramedullary lesion in the terminal spinal cord extending from D12 to L2, resulting in fusiform cord expansion and scalloping of the posterior vertebral margins, likely representing ependymoma or schwannomatous involvement. Optical coherence tomography (OCT) imaging (Fig. 4) demonstrated marked thinning of the peripapillary retinal nerve fiber layer in both eyes, supporting the diagnosis of bilateral optic atrophy. These findings, in conjunction with the clinical picture of bilateral progressive sensorineural hearing loss, optic neuropathy, and a longstanding neck mass, were diagnostic of Neurofibromatosis Type 2. Further evaluations included genetic counseling for NF2 gene mutation testing, neurosurgical consultation for management of the craniospinal tumors, and low vision and audiological rehabilitation planning.

## Discussion

Neurofibromatosis Type 2 (NF2) is an uncommon autosomal dominant tumor-predisposition syndrome caused by pathogenic variants in the *NF2* gene at 22q12.2, encoding the tumor suppressor protein merlin. Approximately half of cases are inherited, while the remainder result from de novo mutations, frequently with somatic mosaicism that complicates molecular confirmation^[8–10]^. The estimated prevalence is 1 in 25 000, and although survival has modestly improved with modern interventions, the median life expectancy remains in the third to fourth decade, largely due to complications from intracranial tumors and cranial neuropathies^[11–13]^. A 10-year survival approximates 67%, with actuarial survival averaging 15 years from diagnosis^[13,14]^.

Our patient demonstrated an unusual triad of bilateral optic neuropathy, profound sensorineural hearing loss, and a chronic cervical mass. The initial differential included demyelinating optic neuritis, compressive meningioma, mitochondrial optic neuropathy, and congenital inner-ear disease. However, MRI revealed bilateral enhancing vestibular schwannomas extending into the internal auditory canals, together with multiple spinal schwannomas – findings pathognomonic of NF2 and satisfying the Manchester diagnostic criteria^[15,16]^. While vestibular schwannomas are nearly universal, NF2 frequently involves additional intracranial and spinal tumors including meningiomas, ependymomas, and peripheral schwannomas^[2,17,18]^. Cutaneous manifestations occur in ~70% of patients, though typically with fewer than 10 lesions^[1]^. Ocular complications – posterior subcapsular cataracts, optic nerve sheath meningiomas, and retinal hamartomas – are equally important contributors to morbidity^[15,16,18,19]^.

Optic neuropathy in NF2 arises through distinct mechanisms. Direct tumor infiltration (optic sheath meningiomas or merlin-deficient gliomas), secondary compression and ischemia from raised intracranial pressure or obstructive hydrocephalus, and chronic axonal loss reflected in retinal nerve fiber layer thinning on OCT all contribute^[16,19]^. In this case, diffuse optic atrophy with concomitant ventriculomegaly suggested a compressive–ischemic etiology, though primary optic nerve sheath involvement could not be excluded.

At the molecular level, NF2 tumorigenesis follows a “two-hit” model, where a germline or mosaic variant is compounded by somatic loss of the second allele. Merlin loss deregulates multiple proliferative cascades, including Ras/MAPK, PI3K/AKT, mTOR, Rac/PAK/JNK, FAK/Src, and Hippo pathways, explaining the diverse tumor spectrum^[1,8,13,14,20–22]^. Genotype–phenotype correlations are increasingly recognized: truncating and N-terminal mutations often result in the severe Wishart phenotype, characterized by early onset, rapid tumor growth, multiple meningiomas, and ocular complications, whereas missense or splice-site variants in the C-terminal region generally present as the milder Gardner phenotype with later onset and slower progression^[10,16,23–25]^.

Management remains complex and multidisciplinary. Microsurgical excision of vestibular schwannomas is associated with high recurrence and significant morbidity, particularly hearing loss and facial nerve palsy^[2,24]^. Stereotactic radiosurgery provides local control in selected cases but increases the risk of neuropathy and complicates future surgery^[20,26–28]^. The therapeutic landscape has shifted with biologic agents: bevacizumab, an anti-VEGF monoclonal antibody, induces tumor shrinkage or stabilization in up to 80–88% of cases, with hearing improvement or stabilization in 60–70%^[29,30]^. However, rebound growth, proteinuria, and hypertension limit long-term use^[29–31]^. Investigational therapies – including mTOR inhibitors (everolimus), tyrosine kinase inhibitors (lapatinib, erlotinib), MEK inhibitors (mirdametinib), and VEGFR peptide vaccines – are under active clinical evaluation, with promising preliminary results^[32]^. For advanced hearing loss, cochlear or auditory brainstem implants provide rehabilitative benefit^[30,32,33]^.

Prognosis in NF2 is determined by genotype, tumor burden, and functional impact. Early-onset, truncating variants confer an aggressive course with widespread tumor load and earlier mortality, whereas mosaic or missense mutations predict milder progression. Morbidity is largely dictated by bilateral vestibular schwannomas, intracranial meningiomas, and spinal ependymomas. Early bilateral hearing loss and optic neuropathy, as in our patient, lead to profound disability and reduced quality of life^[1,5,24,29]^.

Lifelong structured surveillance is essential. Current consensus recommends annual brain MRI with internal auditory canal views, spinal MRI every 1–3 years depending on tumor burden, annual audiometric assessments with speech perception testing, and regular ophthalmic evaluation with slit lamp, OCT, and visual fields^[32–34]^. Genetic counseling remains critical given the 50% familial transmission risk and nearly complete penetrance by midlife. Optimal outcomes require integrated care involving neurosurgery, neuro-oncology, otology, ophthalmology, audiology, rehabilitation, and genetics^[2,8,10,33]^.

Although curative therapy remains elusive, early detection and individualized management strategies – balancing the risks of surgery and radiotherapy against the potential benefits of systemic approaches such as bevacizumab – offer hope for stabilizing disease and preserving function. With advances in genotype-based prognostication and biologic therapies, the outlook for NF2 is gradually shifting from purely palliative care toward proactive, functional preservation^[29,30,32–34]^.

## Conclusion

This case emphasizes the diagnostic challenge of NF2 in young patients with multisystem neurological deficits. Recognition of the characteristic tumor spectrum through MRI is central to diagnosis in resource-limited settings where genetic testing is not always feasible. Multidisciplinary surveillance and individualized therapeutic planning are vital to preserve sensory function and prolong survival. The integration of targeted biologics and genotype-driven risk stratification represents an evolving frontier in NF2 care, gradually shifting the prognosis from inevitably disabling toward one of managed chronicity with preserved function.

## Data Availability

Not applicable.

## Footnotes

All potential sponsorships or competing interests relevant to the content have been disclosed above.
